# Light Stimuli and Circadian Clock Affect Neural Development in *Drosophila melanogaster*

**DOI:** 10.3389/fcell.2021.595754

**Published:** 2021-03-05

**Authors:** Eleni Dapergola, Pamela Menegazzi, Thomas Raabe, Anna Hovhanyan

**Affiliations:** ^1^Institute of Medical Radiation and Cell Research, Biozentrum, University of Würzburg, Würzburg, Germany; ^2^Neurobiology and Genetics, Theodor-Boveri Institute, Biozentrum, University of Würzburg, Würzburg, Germany

**Keywords:** *Drosophila*, neuroblast growth, proliferation, circadian clock, light stimuli

## Abstract

Endogenous clocks enable organisms to adapt cellular processes, physiology, and behavior to daily variation in environmental conditions. Metabolic processes in cyanobacteria to humans are under the influence of the circadian clock, and dysregulation of the circadian clock causes metabolic disorders. In mouse and *Drosophila*, the circadian clock influences translation of factors involved in ribosome biogenesis and synchronizes protein synthesis. Notably, nutrition signals are mediated by the insulin receptor/target of rapamycin (InR/TOR) pathways to regulate cellular metabolism and growth. However, the role of the circadian clock in *Drosophila* brain development and the potential impact of clock impairment on neural circuit formation and function is less understood. Here we demonstrate that changes in light stimuli or disruption of the molecular circadian clock cause a defect in neural stem cell growth and proliferation. Moreover, we show that disturbed cell growth and proliferation are accompanied by reduced nucleolar size indicative of impaired ribosomal biogenesis. Further, we define that light and clock independently affect the InR/TOR growth regulatory pathway due to the effect on regulators of protein biosynthesis. Altogether, these data suggest that alterations in InR/TOR signaling induced by changes in light conditions or disruption of the molecular clock have an impact on growth and proliferation properties of neural stem cells in the developing *Drosophila* brain.

## Introduction

Endogenous circadian clocks are highly conserved and enable organisms to adjust their physiology and behavior to the day/night cycle. All circadian clocks (1) synchronize to the environment through input pathways, (2) rely on molecular oscillators, which generate the rhythm and thereby keep circadian time, and (3) transmit time information to modulate behavior and physiology through output pathways. Processes modulated by the circadian clock include feeding behavior, locomotor activity, body temperature, hormone level, and metabolic activity (reviewed in Green et al., 2008; Allada and Chung, 2010; Dubowy and Sehgal, 2017).

A hierarchical network of clocks located in different tissues controls these rhythmic processes. The master clock is located in the central nervous system (CNS) and synchronizes organ and tissue clocks. The mammalian master clock resides in the suprachiasmatic nuclei of the hypothalamus comprising about 15,000 clock neurons, whereas in *Drosophila*, the master clock consists of about 150 clock neurons allocated in several clusters in the lateral and dorsal brain (reviewed in Glossop and Hardin, 2002; Hermann-Luibl and Helfrich-Förster, 2015; Patke et al., 2020). The molecular clock machinery is largely conserved across different species and consists of transcriptional-translational feedback loops to maintain the rhythmic cycling of gene expression (reviewed in Patke et al., 2020). Briefly, circadian activators trigger transcription of repressor genes, which, upon translation, feedback to suppress their own transcription. In *Drosophila*, these circadian activators are Clock (Clk) and Cycle (Cyc), which form a heterodimeric protein complex to trigger the transcription of the circadian repressors, *period* (*per*) and *timeless* (*tim*), as well as many other target genes. A complex interplay of light-dependent degradation, initiated by the blue light photoreceptor Cryptochrome (Cry), and multiple phosphorylation events regulate the accumulation of Per/Tim heterodimers and their timely translocation into the nucleus to inhibit the transcriptional activity of Clk/Cyc (reviewed in Hardin and Panda, 2013; Dubowy and Sehgal, 2017; Patke et al., 2020). Most core clock components are transcriptional regulators, regulating the expression of approximately 10% of all genes in a circadian manner (reviewed in Wijnen et al., 2006; Doherty and Kay, 2010; Hughes et al., 2012). Therefore, dysregulation of the circadian system contributes to the pathophysiology of many diseases; most prominent of those are psychiatric disorders and metabolic diseases (reviewed in Bellet and Sassone-Corsi, 2010; Zordan and Sandrelli, 2015; Maury, 2019).

The larval brain clock network is much simpler organized with only nine clock neurons in each hemisphere: five lateral neurons (LNs) and two pairs of dorsal neurons (DN1s and DN2s) (Kaneko et al., 1997). Light sensing is achieved by Cry expression in four out of the five LNs and the DN1s as well as from the larval visual system, the Bolwig organ (BO). The 12 photoreceptor cells of the BO project are divided into two subtypes according to either rhodopsin 6 (Rh6, sensitive to green light) or rhodopsin 5 (Rh5, sensitive to blue light) expression (Malpel et al., 2002; Mazzoni et al., 2005). They project to the four Cry^+^ LNs that act as early pacemaker neurons (PNs), which are responsible for synchronization of the larval brain clock to the light-dark (LD) cycle. Rh5, Rh6, and Cry are each sufficient alone to entrain all larval clock neurons (Keene et al., 2011; Klarsfeld et al., 2011). Rhythmic Per expression in these early PNs throughout development indicated a functional clock network before other clock neurons are integrated at late developmental stages (Kaneko et al., 1997; Liu et al., 2015). Cycling of Per in early PNs is maintained under DD conditions with the peak time of Per expression comparable to LD conditions, indicating a free-running period of approximately 24 h (Kaneko et al., 1997). Despite the early appearance of a functional clock, no clock-controlled behavior has been characterized in larvae. A well-defined light-dependent larval behavior is negative phototaxis. Although light avoidance is not regulated in a circadian manner, it requires Rh5 expressing photoreceptors, DN2s, and a single LN (Keene et al., 2011). Even though light during development is dispensable for circadian behavior of individual adults, a single light pulse at larval stages is sufficient to synchronize the activity phases of adult flies reared under constant darkness (Sehgal et al., 1992). A similar effect was observed when the light pulse was given during the second half of embryogenesis (Zhao et al., 2019). Thus, clock neurons in larvae fulfill distinct functions.

Whereas the clock network in the *Drosophila* CNS regulates circadian rhythms such as locomotor activity, sleep, and eclosion, circadian control of metabolism mostly depends on peripheral oscillators (reviewed in Green et al., 2008; Allada and Chung, 2010). One of the major peripheral clocks in mammals is placed in the liver, which regulates among others metabolism by combining environmental and central clock signals (Lamia et al., 2008). However, limited food conditions might set the peripheral clock without any involvement of the master clock (Damiola et al., 2000; Hara et al., 2001). In *Drosophila*, the fat body takes over the liver function to regulate feeding behavior and nutrient storage (Xu et al., 2008). In response to nutritional cues, the larval fat body generates mitogens to promote cell growth. A prominent example of fat body dependent cellular growth are the neural progenitor cells (neuroblasts, NBs) in the developing CNS (reviewed in Homem and Knoblich, 2012; Hakes and Brand, 2019). Three different types of NBs exist in the CNS, which differ by their division modes: Type 0, Type I, and Type II (reviewed in Doe, 2017; Hakes and Brand, 2019). Similar to other stem cells, NBs divide asymmetrically to self-renew and to generate a daughter cell. Daughter cells generated by Type 0 NBs directly differentiate into neurons. Division of Type I NBs gives rise to ganglion mother cells (GMCs), which divide once more to generate two neurons. Type II NBs generate intermediate neural progenitors (INPs), which in turn produce GMCs.

Two waves of neurogenesis, embryonic and larval, ensure CNS development and both correlate with changes in neuroblast (NB) size (reviewed in Homem and Knoblich, 2012; Yasugi and Nishimura, 2016). During the embryonic phase, NBs go through a limited number of divisions, diminishing in size with each division until they become quiescent. The four mushroom body (MB) NBs are exceptional because they proliferate throughout development without a quiescence phase (Ito and Hotta, 1992) to generate the neurons of the MBs, important structures for the learning and memory processes (reviewed in Heisenberg, 2003; Cognigni et al., 2018). The second wave of neurogenesis, during larval and pupal stages, starts with the exit of NBs from quiescence in a nutrition-dependent manner (Britton and Edgar, 1998; Chell and Brand, 2010). In contrast to the embryonic NBs, the larval NBs maintain their original size by re-growing after each division. This continues until the end of neurogenesis, where again NBs decrease in size and exit from the cell cycle (Siegrist et al., 2010).

The major growth regulator pathway to trigger exit from NB quiescence and reactivation of proliferation is governed by insulin receptor (InR)/target of rapamycin (TOR) signaling. The pathway is stimulated by insulin-like peptides (ILPs) generated in insulin-producing glial cells, which receive nutritional signals from the fat body (Géminard et al., 2009; Chell and Brand, 2010; Sousa-Nunes et al., 2011). The TOR pathway might be activated independently from the InR pathway via direct cellular nutrient sensing (reviewed in González and Hall, 2017). The interplay between InR and TOR pathways regulates cell growth through a variety of effector proteins at the levels of gene expression, ribosome biogenesis, and protein synthesis (reviewed in Hietakangas and Cohen, 2009; Russell et al., 2011).

Despite the well-established link between the circadian system and physiological processes, the influence of the circadian clock on neuronal development and therefore its potential impact on neural circuit formation is poorly understood. To investigate such a link, we compared wild-type and clock mutant larvae exposed to different light regimes. First, we showed that disruption of the molecular circadian clock and different light conditions affect NB growth and proliferation. Second, we found a significant reduction of nucleolus size in NBs, indicating that rRNA production and ribosome biogenesis might be disturbed. Based on these findings we analyzed the effect of light and the circadian clock on the InR/TOR growth regulatory pathway. Specifically, gene expression and activity of Akt as a downstream effector kinase of InR signaling, and S6K (RPS6-p70-protein kinase) as a target of TOR and major regulator of nutrient-dependent metabolism and cell growth, were disturbed when the light regime was changed or clock function was abrogated.

## Materials and Methods

### Fly Stocks and Genetics

Flies were maintained at 25°C on standard cornmeal food in a 12 h light-dark (LD) cycle. *Canton Special* (*CS*) was the control and genetic background for *cry*^01^ (Dolezelova et al., 2007) and clock mutant flies: *per*^01^ (Konopka and Benzer, 1971). To mark Type II NBs, the *wor-Gal4*, *ase-Gal80* driver line was used to express *UAS-mCD8:GFP* (Neumüller et al., 2011). For RNA isolation or protein extraction, after laying eggs, animals were entrained in a 12 h LD cycle or shifted into constant darkness until the 3rd instar larval stage. Relative to Zeitgeber time 0 (ZT0) as the time of lights-on during the LD cycle and circadian time 0 (CT0) as the time corresponding to subjective lights-on during free running in DD, larvae were collected in a ZT0-ZT4-ZT8-ZT12-ZT16-ZT20 or CT0-CT4-CT8-CT12-CT16-CT20 schedule and dissected. In the case of *per*^01^ larvae were always collected at ZT0 and dissected. Isolated larval brains were collected either in TRIzol (for RNA extraction) or in Laemmli buffer (for protein extraction).

### Larval Staging

Immediately after hatching, larvae were collected from apple juice plates at 30 min intervals and transferred to standard fly food plates with yeast. *per*^01^ mutants, wild-type larvae under LD and DD light regime, were kept at 25°C until they reached the desired age. Before preparation, larval stages were determined by means of spiracle morphology. Preparation of 1st instar larvae (L1) was done 22 h after larval hatching (ALH), 2nd instar larvae (L2) were 36 h ALH, and 3rd instar larvae (L3) were at the wandering stage approximately 120 h ALH.

### Immunohistochemistry

For immunostainings, larval brains were dissected in PBS (10 mM Na_2_HPO_4_,2 mM KH_2_PO_4_, 2.7 mM KCl, 137 mM NaCl) and fixed on ice for 25 min in PLP solution (2% paraformaldehyde, 10 mM NaIO_4_, 75 mM lysine, 30 mM sodium phosphate buffer, pH 6.8). All washings were done in PBT (PBS plus 0.3% Triton X-100). After blocking in PBT containing 3% normal goat serum for 2 hr, brains were incubated overnight with combinations of the following primary antibodies: rabbit anti-protein kinase C (anti-PKC) (1:1000; clone C20, Santa Cruz Biotechnology, Dallas, TX, United States), rabbit anti-phospho-histone H3 (1:2500; Merck Millipore, Burlington, MA, United States), rat anti-Miranda (1:300; clone CD#5-7E9BG5AF4, Abcam, Cambridge, United Kingdom), mouse anti-Miranda (1:20; F. Matsuzaki, Kobe, Japan), rabbit anti-Nop5 (1:600; G. Vorbrüggen, Göttingen, Germany), chicken anti-GFP (1:1000; Abcam), rabbit anti-Asense (1:400; F. Diaz-Benjumea, Madrid, Spain), guinea pig anti-Deadpan (1:1000; J. Knoblich, Vienna, Austria), mouse anti-Dachshund (1:10; clone mAbdac2-3, Developmental Studies Hybridoma Bank [DSHB], Iowa City, IA, United States), rabbit anti-Tailless (1:600; J. Reinitz, Chicago, Illinois, United States), guinea pig anti-lamin DmO (1:300; G. Krohne, Würzburg, Germany), mouse anti-Bruchpilot (1:30; clone nc82, DSHB). Alexa Fluor 546-Phalloidin (1:100; Molecular Probes, Thermo Fisher Scientific, Waltham, MA, United States) was used to mark F-actin. Secondary antibodies were conjugated with Alexa Fluor 488 (Molecular Probes), Cy3, Cy5, or DyLyte488 (Dianova, Hamburg, Germany). After extensive washing in PBT, brains were embedded in Vectashield using distance holders to avoid brain deformation. Confocal images were collected with a Leica SPE microscope. Image processing was done with ImageJ 1.46r software (NIH, Bethesda, MA, United States).

Neuroblasts were visualized by aPKC or Miranda, nucleoli by Nop5 antibodies. The freehand selection tool of ImageJ 1.46r software was used for measuring NB and nucleolar areas. This allowed to include spherical shaped metaphase NB and the more irregular shaped NB at other cell cycle stages in our analysis. Data were blinded and analyzed independently by at least two persons.

To determine the brain volume of 3rd instar larvae (120 h ALH), the average brain lobe diameter was calculated from two orthogonal diameters taken across the brain lobe at its largest dimension, and volume (V) was calculated as *V* = 4/3πr^3^. Brain volume was the sum of volumes of the two brain lobes. Adult central brain (CB), optic lobe (OL), and whole brain size measurements of the different genotypes were scored blind with the freehand selection tool of ImageJ 1.46r software.

### Neuroblast Proliferation

The anti-Tailless antibody was used to specifically mark MB NB and derived GMCs (Kurusu et al., 2009). Type II NBs and their lineages were marked with GFP by using *UAS-mCD8:GFP* line under the control of the *wor-*Gal4, *ase-*Gal80 driver line (Neumüller et al., 2011). To distinguish different maturation stages of INPs within the Type II NB lineage, anti-Dpn and anti-Ase antibodies were used (Bowman et al., 2008; Walsh and Doe, 2017). MB NB derived GMCs and mature INPs (mINPs) were counted with the Amira^®^ software using the Landmark selection.

### RNA Isolation and RT-qPCR Analysis

Wild type and *per*^01^ mutant larvae were collected in PBS and placed on ice. Dissections of larvae were done within 30 min and brains were collected in TRIzol^®^ reagent for total RNA extraction according to the manufacturer’s instruction (Ambion^®^, Thermo Fisher Scientific, Waltham, MA, United States). First-strand cDNA was synthesized from 2 μg of RNA using High-Capacity cDNA Reverse Transcription Kits (Applied Biosystems, Thermo Fisher Scientific, Waltham, MA, United States). RT-qPCR was done using PowerUp^TM^ SYBR^TM^ Green Master Mix (Applied Biosystems) on a StepOnePlus^TM^ (Applied Biosystems) real-time thermal cycler. Reaction mixtures contained 300 nM of oligonucleotides. RT-qPCR conditions were 2 min 50°C and 2 min 95°C holding steps, followed by 40 cycles of 15 s 95°C and 1 min 60°C. Results were expressed as fold change in expression of the treated sample in relation to untreated samples and relative to the reference gene *rp49*. Mean ± SEM was calculated at least from triplicate experiments from each of the three independent biological samples per genotype or different light regime.

The following primers were used to amplify the cDNA of target genes:

*Akt:* forward 5′-ACAGATCTAGTGTTGAAAAAAATATA CCG-3′

reverse 5′-ATGTCTCCTTGGTAGCTGAACTGCG-3′,

*S6k:* forward 5′-TTCTTAGAGGATACCACATGCTTC-3′

reverse 5′-TGGTCAAAATTTCAGGTGCCATGTAC-3′,

*rp49:* forward 5′-GCCCAAGATCGTGAAGAAGC-3′

reverse 5′-CGACGCACTCTGTTGTCG-3′.

### Western Blot

Lysates from wild-type or *per*^01^ larval brains were sonicated in 2x Laemmli, separated by SDS-PAGE and transferred to PVDF membranes (Amersham^TM^ Hybond^TM^ P 0.45 PVDF, GE Healthcare Life science, Chicago, IL, United States). Blots were incubated overnight at 4°C with the following antibodies: rabbit anti-phospho-Akt (Ser473) (1:1000; clone D9E, Cell Signaling, Danvers, MA, United States), rabbit anti-Akt (1:1000, Cell Signaling), rabbit anti-phospho-Drosophila-p70 S6k (1:1000; Cell Signaling), mouse anti-α-Tubulin (1:5000, clone NDM1A, Merck, Darmstadt, Germany). After incubation with HRP-coupled secondary antibodies, signal detection was done with the ECL Plus detection reagents (GE Healthcare Life Science, Buckinghamshire, United Kingdom) and a ChemoCam ECL Imager equipped with a 16 bit camera (Intas, Göttingen, Germany). Exposure times were adjusted to allow for quantification of signal intensities within the dynamic range of the camera system.

### Data Analysis

Nucleolar size, cell size, and number of progenies were analyzed by Statistica *v*. 9. Distributions of variables did not deviate significantly from normality (Kolmogorov-Smirnov test; *P* > 0.2). A one-way analysis of variance (ANOVA) was performed for statistical analysis. Axis lengths and areas were considered as dependent variables, and the strain (wild type versus mutant or LD light regime versus DD) was considered as an independent variable.

To calculate the significance of gene expression differences between experimental groups, statistical analyses were performed using the Mann–Whitney-*U*-test (Origin Pro9.0.0 b45 software). For multiple testing within one data set, the level of significance was adjusted with the Bonferroni correction factor. Graphs are presented as mean ± the max and min size distribution or ± SEM, asterisks depict the level of statistical significance ^****^p ≤ 0.00001 and ^∗∗∗^*p* ≤ 0.0001, ^∗∗^*p* ≤ 0.001, and ^∗^*p* ≤ 0.01. Graphs were generated in Prism 6.

## Results

### Circadian Clock and Light Independently Control Cell Growth in the Larval Brain

**FIGURE 1:**
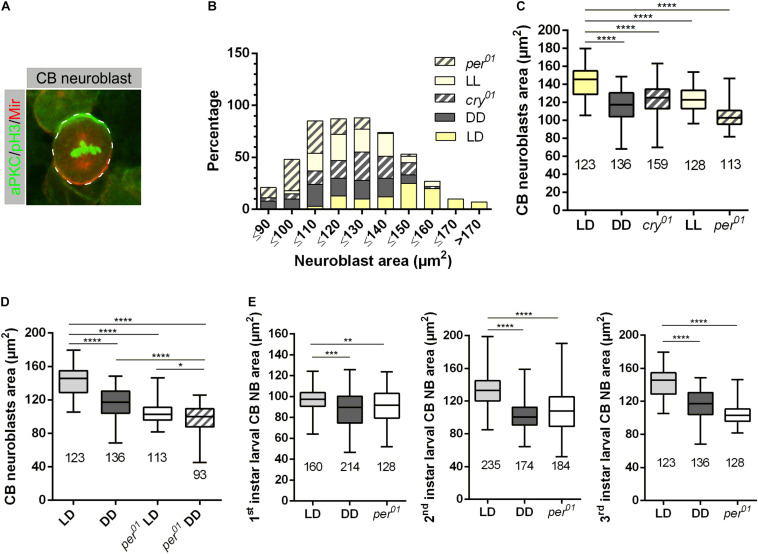
Light and the circadian clock control neuroblast size. **(A)** Representative example of a central brain (CB) neuroblast (NB) labeled with aPKC (green) and Miranda (red) and phospho-histone H3 (pH3, green) as a mitotic chromatin marker. **(B)** CB NBs size distribution of wild-type larvae grown under 12:12 h dark/light (LD), constant light (LL), and constant darkness (DD). LD conditions were used for *cry*^01^ and *per*^01^ mutant animals. NBs in wild type mainly distribute within 120 to 170 μm^2^, NBs in wild-type larvae (DD) and *per*^01^ mutants range between 90 and 140 μm^2^, whereas NB cell size for larvae grown under LL condition and *cry*^01^ distribute between 110 and 150 μm^2^. The number of measured NB for each genotype and light conditions are indicated in **(C)**. **(C)** Average NBs sizes of all experimental groups shown in **(B)** (^****^p < 0.00001). **(D)** The NB size defect in *per*^01^ mutant larvae is enhanced under DD conditions (^****^p < 0.00001, ^∗^*p* < 0.01). **(E)** NB size defects are observed throughout larval development for wild type (DD) and *per*^01^ (^****^p < 0.00001, ^∗∗∗^*p* < 0.0001, ^∗∗^*p* < 0.001). At least 10 brains were analyzed for each genotype and different light condition. The number of measured NBs are indicated below each box plot.

At all developmental stages, animals sense and respond to changes in both external and internal conditions. The combination of this information regulates behavior and metabolism to benefit from available resources and to maintain cellular homeostasis. Metabolism is important for the proper growth and development of an organism (reviewed in Koyama et al., 2020) and many aspects of metabolism and cellular physiology are controlled by endogenous circadian rhythms (reviewed in Green et al., 2008; Bellet and Sassone-Corsi, 2010; Shi and Zheng, 2013). CNS development of *Drosophila* is a prominent example to investigate mechanisms, how an organism copes with changes in environmental conditions, e.g., the nutritional status. Nutritional signals after larval hatching are important for NB growth and reactivation of proliferation after the quiescence phase, whereas reduction in NB growth and proliferation terminates neurogenesis during early pupal stages (Siegrist et al., 2010; Spéder and Brand, 2014; reviewed in Lanet and Maurange, 2014). To look for a potential impact of the circadian clock on NB growth, we investigated whether disruption of the circadian clock influences the size of central brain (CB) NBs during development. Moreover, we asked whether light, independently from the clock, also affects cell growth. We measured NB sizes in 3rd instar larval brains of wild type and *per*^01^ mutants (which lack a functional clock) grown under light-dark (LD) condition. NBs were marked with specific markers (aPKC and Miranda, Figure 1A). Wild type flies were also kept in constant darkness (DD, where the molecular clock still maintains a rhythm of approximately 24 h without being reset by light stimuli) and constant light (LL, where the circadian clock is not functional anymore due to the constant activation of the photopigment Cry). In addition, to control for effects of the light-dark cycles independent of resetting of the molecular oscillator by Cry, we included *cry*^01^ mutants in our experiments. We found significantly smaller NBs in larvae lacking a functional clock, namely *per*^01^ mutants and wild type kept under LL, respectively, DD conditions, as well as in larvae with a functional oscillator but unable to reset it by light transitions (*cry*^01^ mutants) (Figures 1B,C). If light and clock independently affect cell growth, the combination of clock disruption and absence of light should enhance the observed cell size defect. Therefore, we analyzed CB NB sizes from *per*^01^ mutant 3rd instar larvae kept under DD conditions. Indeed, the average size of NBs was further reduced in comparison to either *per*^01^ or wild-type larvae kept in DD, although reduction was neither synergistic nor additive (Figure 1D). In combination with the NB size reduction observed in *cry*^01^ mutants and animals kept under LL condition, this indicated at least partial independent effects of light and clock on cell growth. As the strongest effects were observed in *per*^01^ mutants and wild type grown under DD condition, all further experiments were performed using these two experimental groups.

In order to distinguish whether the observed growth defect exists from the beginning of the second wave of neurogenesis starting with larval hatching or it is a later developmental effect, we conducted a staging experiment by measuring the size of NBs at 22 h (1st instar), 36 h (2nd instar), and 120 h (3rd instar) after larval hatching (ALH). The late 1st instar larval stage was chosen because NBs are sequentially released the quiescence phase starting in early 1st instar larvae (Ito and Hotta, 1992). A significant NB cell size defect was already observed in late 1st instar larvae, which became much more pronounced at later larval stages (Figure 1E).

**FIGURE 2:**
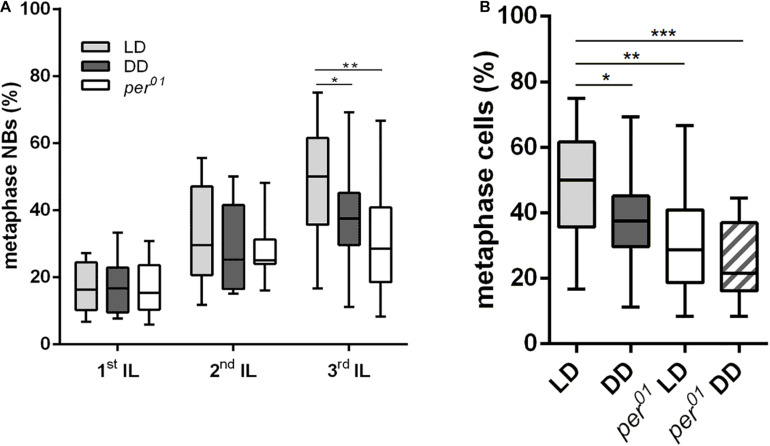
Effects of the circadian clock and light on neuroblast division rates. **(A)** The number of metaphase NB in relation to the overall NB number was determined in 1st, 2nd, and 3rd instar larval (IL) central brains. Compared to wild type (LD), the DD condition and the *per*^01^ mutant caused a significant reduction of dividing NBs only in 3rd instar larval brains (^∗^*p* < 0.01, ^∗∗^*p* < 0.001). **(B)** The number of metaphase NBs in 3rd instar larval brains was further decreased when *per*^01^ larvae were kept in DD (^∗∗∗^*p* < 0.0001). At least 16 brains were analyzed for each larval stage and experimental condition.

Since NB reactivation in early 1st instar larvae is accompanied by cell growth as a prerequisite for proliferation, the observed cell size defect could be a consequence of a delay in reactivation. Quiescent NBs are arrested either in G0 or in G2 phase of the cell cycle. Within a time window of 48 h ALH, NBs arrested in the G2 stage reactivate earlier compared to G0 NBs (Otsuki and Brand, 2018). The percentage of metaphase NBs was used as a proxy for proliferation activity of the whole NB population. Whereas the number of metaphase NBs in 1st (22 h ALH) and 2nd (36 h ALH) instar larvae were comparable between wild type animals (LD and DD conditions) and the *per*^01^ mutant, we observed a 20–30% reduction in the number of metaphase NBs in 3rd instar larval brains (120 h ALH) from wild type (DD) and the *per*^01^ mutant (Figure 2A). This effect was further enhanced when *per*^01^ animals were kept in DD (Figure 2B). Thus, NB which became reactivated until 36 h ALH started to proliferate in a wild type manner in both experimental groups.

Given the pronounced NB cell size defect at all larval stages, the question arises whether animals with a disrupted clock or grown under DD condition show a developmental delay at the organismic level. Using the hatching of 1st instar larvae as a starting time point, it became evident that pupation time points for all genotypes are largely comparable, with a minor delay of DD kept animals (Supplementary Figure 1). Correspondingly, there was no obvious difference in the appearance of wandering 3rd instar larvae for wild type and *per*^01^, but an approximately 1-day delay in the appearance of wild type larvae grown under DD conditions (data not shown).

In summary, our findings provided evidence that not only a functional endogenous clock but also light as an environmental factor are needed to control proper NB size, which correlates with impaired proliferation at the end of larval life.

### Circadian Clock and Light Are Required for Cell Proliferation

**FIGURE 3:**
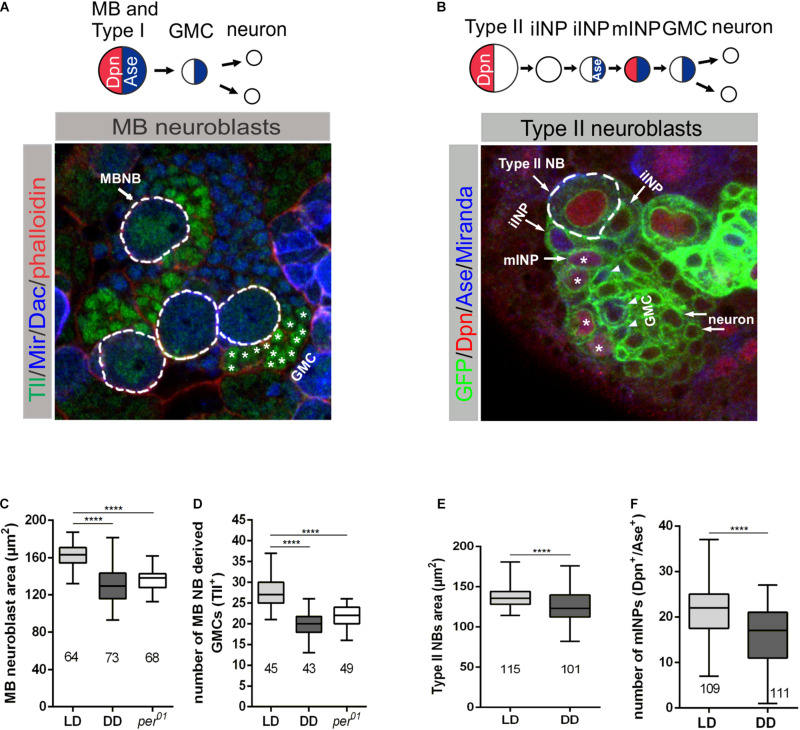
Effect of light and the molecular clock on mushroom body and Type II neuroblast sizes and proliferation. **(A)** Miranda (Mir blue) was used as a NB marker, Tailless (Tll green) marks mushroom body (MB) NBs and derived GMCs (*), Dachshund (Dac blue) labels MB neurons in wandering 3rd instar larval brains. Phalloidin (red) was used as a marker for cortical actin. **(C)** The decreased MB NB size under DD conditions and in *per*^01^ mutant larvae was accompanied by a reduced number of GMCs **(D)**. **(B)** Type II NB lineages were marked with GFP (green) expressed under *wor-Gal4*, *ase-Gal80* control. In addition to the cortical NB marker Miranda (blue), nuclear Dpn (red) and Ase (blue) were used to distinguish immature INPs (iINP) and mature INPs (mINP*). **(E)** Type II NB size as well as the number of mINPs (Dpn^+^/Ase^+^) **(F)** were significantly reduced in larvae under DD conditions. At least 10 brains were analyzed for each genotype or light condition. Data represent the mean obtained from the number of measured NBs or counted progenies ± the max and min size distribution. The number of measured NBs or progeny cells are indicated below of each box plot (^****^p < 0.00001).

The coordination between cell growth and proliferation is important for the proper development of tissues (reviewed in Homem et al., 2015). Since we found evidence that the endogenous clock and light have an impact on the growth of NBs, we assumed that the generation of progeny cells by NBs might be affected by the circadian clock and light. Because this issue is difficult to address for the whole heterogeneous NB population, we determined the number of progeny cells for two well-characterized subtypes of NBs: mushroom body (MB) NBs and Type II NBs. The number of these NBs are only four, respectively, eight in each hemisphere, and specific markers are available to label these NBs and their progeny cells. MB and Type II NBs have different modes of division. MB NBs follow the Type I NB division mode to give rise to a self-renewing NB and a GMC, which divides one more time to generate two neurons (Figure 3A). Type II NBs divide asymmetrically to generate a self-renewing NB and a transient amplifying cell called immature intermediate neural progenitor (iINP), which by transcriptional changes becomes a mature INP (mINP). Each mINP divides asymmetrically three to five times to form another mINP and a GMC, giving rise to two neurons (Figure 3B). To identify MB NBs and their progenies, in addition to Miranda (Mir) as a general NB marker, an anti-Tailless (Tll) antibody was used to label MB NBs and derived GMCs, whereas Dachshund (Dac) is expressed in MB neurons (Figure 3A; Kurusu et al., 2000, 2009; Kraft et al., 2016). To label the Type II NB lineage, *UAS-mCD8:GFP* was expressed under the control of *wor-Gal4*, *ase-Gal80* (Neumüller et al., 2011). Additionally, anti-Deadpan (Dpn) and anti-Asense (Ase) antibodies were used to distinguish between progenies of Type II NBs, as Dpn is expressed only in Type II NBs and mINPs, whereas Ase labels iINPs, GMCs and is also co-expressed with Dpn in mINPs (Figure 3B; Bowman et al., 2008; Walsh and Doe, 2017). First, we verified whether the observed general NB growth defect is also evident for MB and Type II NBs. Cell size measurements revealed that both types of NBs in 3rd instar larval brains were significantly reduced in size under DD conditions and in *per*^01^ animals when compared to the wild type LD control (Figures 3C,E). This finding supports our conclusion that light input and a functional clock are required for all NBs to maintain proper cell size. To determine whether the observed reduced cell size is restricted to the NB population, we also examined the cell sizes of MB NB derived GMCs and Type II NB derived mINPs. The size of the analyzed progenies was comparable among the different experimental groups (Supplementary Figure 2).

For analysis of the proliferation capacity of MB or Type II NBs, we counted Tll^+^ GMCs and Dpn^+^/Ase^+^ mINPs generated from single NBs, respectively. In both cases, we observed a pronounced reduction in the number of GMCs and mINPs under DD conditions and in *per*^01^ animals (Figures 3D,F).

Since the GMCs and INPs are the precursors for neurons, one consequence could be a reduced brain size. To address this question, 3rd instar larval brain volumes were determined from stainings with antibodies against the synaptic protein Bruchpilot (Brp) to label neuropile structures and against the nuclear membrane protein Lamin to outline the cellular cortex. We observed a significant reduction in brain size for wild type larvae grown under DD conditions, but not for the *per*^01^ mutant, when compared to brains from wild type (LD) (Supplementary Figure 3A). For the DD condition, this corresponded to the reduced number of progeny cells derived from MB and Type II NBs and also indicated a more general effect on proliferation of NBs. To determine whether DD conditions also have an impact on final brain size in the adult, we measured the area of the CB, the optic lobes (OL), and the complete brain. Brains were stained for Brp and Lamin and reconstructed from optical sections before outlining the borders. In contrast to the analysis of larval brains, DD conditions had no negative influence on adult brain size, which was comparable to wild type (LD) and *per*^01^ mutant brains (Supplementary Figure 3B). Thus, animals can cope at least to a large degree with changes of light conditions or a non-functional clock to reach normal adult brain size despite a defect at the level of NBs.

### Effect of Endogenous Clock and Light on Nucleolar Size and Transcriptional Activity

**FIGURE 4:**
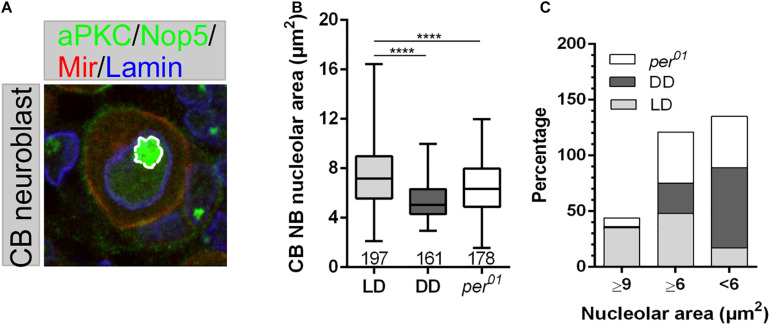
Nucleolar size is affected by light and the circadian clock. **(A)** Neuroblasts in 3rd instar larval brains were marked for cortical aPKC (green) and Mir (red), the nuclear membrane protein Lamin (blue), and Nop5 (green) as a nucleolar marker. **(B)** Compared to the control (wild type, LD), central brain NB nucleolar sizes are significantly reduced under DD conditions and in *per*^01^ animals (^****^p < 0.00001). At least 10 brains were analyzed for each genotype and different light condition. Data represent the mean obtained from the number of measured nucleoli ± the max and min size distribution. The number of analyzed NB are indicated below of each box plot; **(C)** Nucleolar size distribution of the experimental groups shown in **(B)**.

A possible explanation for the compromised proliferation observed for MB and Type II NBs (Figures 3D,F) could be the inability of NBs to produce enough proteins to fulfill the requirements of the cell to gain appropriate mass and size before division. The efficiency of protein biosynthesis depends on proper nucleolar function as a site for rRNA transcription/processing and assembly of ribosomes. Thus, the nucleolus is a critical player to maintain cell homeostasis and directly affects cell growth and proliferation. Nucleolar size positively correlates with the amount of rRNA biosynthesis (reviewed in Stȩpiński, 2018). Specifically, it is directly related to RNA polymerase I transcriptional activity and nucleolar size is highest at the end of the G2 phase, before cell division takes place (Maszewski and Kwiatkowska, 1984, reviewed in Hernandez-Verdun, 2011). To verify, whether light or a disturbed circadian clock influence RNA synthesis, we compared the size of NBs nucleoli in *per*^01^ mutant and wild type grown under DD and LD conditions. Interphase NBs were marked using aPKC and Mir, Lamin outlines the nuclear membrane, and Nop5 was used as a nucleolar marker (Figure 4A).

Nucleolar sizes of NBs in *per*^01^ mutant and wild-type larvae grown under DD conditions were significantly smaller compared to wild type (Figure 4B). Moreover, classification of nucleolar size as large (≥9 μm^2^), intermediate (≥6 μm^2^), or small (<6 μm^2^) revealed the strongest effect in wild-type larvae grown under DD conditions, where 72% of the nucleoli were small and only 27% of them reached intermediate size. In *per*^01^ mutants, NB nucleolar sizes were equally (46%) distributed between intermediate and small sizes, whereas the nucleolar size of wild type in LD conditions was mostly distributed between large (42%) and intermediate (48%), with only 10% nucleoli being small (Figure 4C).

Taken together, these results provided evidence that the capability of NBs of *per*^01^ mutants and wild-type animals grown under DD to synthesize proteins might be impaired, which finally could result in the observed cell growth defects.

### InR and TOR Signaling Are Controlled by the Light Regime and the Circadian Clock

Reactivation of NBs after quiescence and their regrowth after each division during larval development requires the InR/TOR signaling pathways (Chell and Brand, 2010; Sousa-Nunes et al., 2011). In general, impaired InR/TOR signaling results in reduced cell size and proliferation (Zhang et al., 2000, reviewed in Russell et al., 2011). Regulation of protein kinase p70 S6K (S6K) catalytic activity, a downstream target of the TOR pathway, is a major mechanism to control metabolism and cell growth due to its role in ribosome biogenesis and protein translation. Protein kinase Akt is an important signaling molecule in the InR pathway and one upstream regulator of TOR (reviewed in Hietakangas and Cohen, 2009; Russell et al., 2011).

**FIGURE 5:**
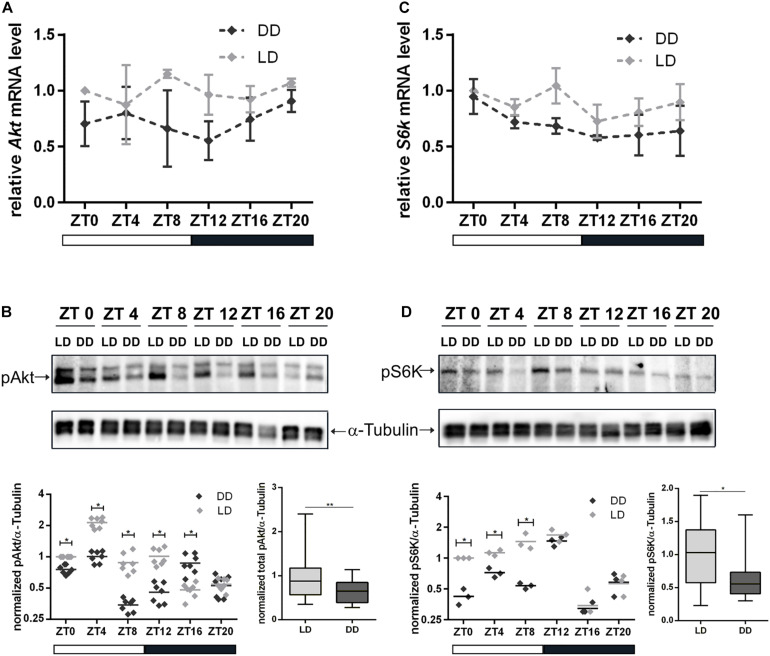
Influence of light on transcription and activation of Akt and S6K. Total RNA and proteins were isolated from wild type 3rd instar larval brains at the indicated Zeitgeber time (ZT). Larvae were grown under LD or DD conditions. **(A,C)** Gene expression of *Akt* **(A)** and *S6k* **(C)** at the indicated ZT and light condition. Each time point represents the mean ± standard error of the mean (SEM) obtained from three biological replicates, each repeated in triplicates. As an internal control, *rp49* was used. **(B,D)** Representative Western blots probed for pAkt **(B)** and pS6K **(D)** at the indicated ZT and light condition. α-Tubulin was used as a loading control. Dot plots show measurements of Akt and S6K phosphorylation level out of seven and three biological replicates, respectively, normalized to α-Tubulin. Box plots display the sum of all time points measurements of Akt and S6K phosphorylation for LD and DD conditions (^∗∗^*p* < 0.001 and ^∗^*p* < 0.01).

Our findings with regard to NB and nucleolar size reduction and the role of nucleolus in the maintenance of cell homeostasis led to the assumption that light might have a regulatory effect on InR/TOR signaling. Therefore, we tested the impact of light on transcriptional and post-transcriptional regulation of Akt and S6K in 3rd instar larval brains. Quantitative reverse transcription (RT)-PCR analyses showed that *Akt* and *S6k* mRNA transcriptional levels were reduced at most time points in DD compared to LD conditions, but differences were below significant levels (Figures 5A,C). Correspondingly, we did not observe any differences for Akt protein total expression level during a period of 24 h between the two experimental groups (Supplementary Figure 4). For S6K, no antibody was available to test for total protein expression in *Drosophila*. Although there was no change at transcriptional level for both kinases and total protein expression level at least for Akt, significant differences were evident in phosphorylation of Akt and S6K at most time points under DD conditions. Both kinases showed a significant reduction in phosphorylation of residues as a readout of kinase activity (Figures 5B,D).

**FIGURE 6:**
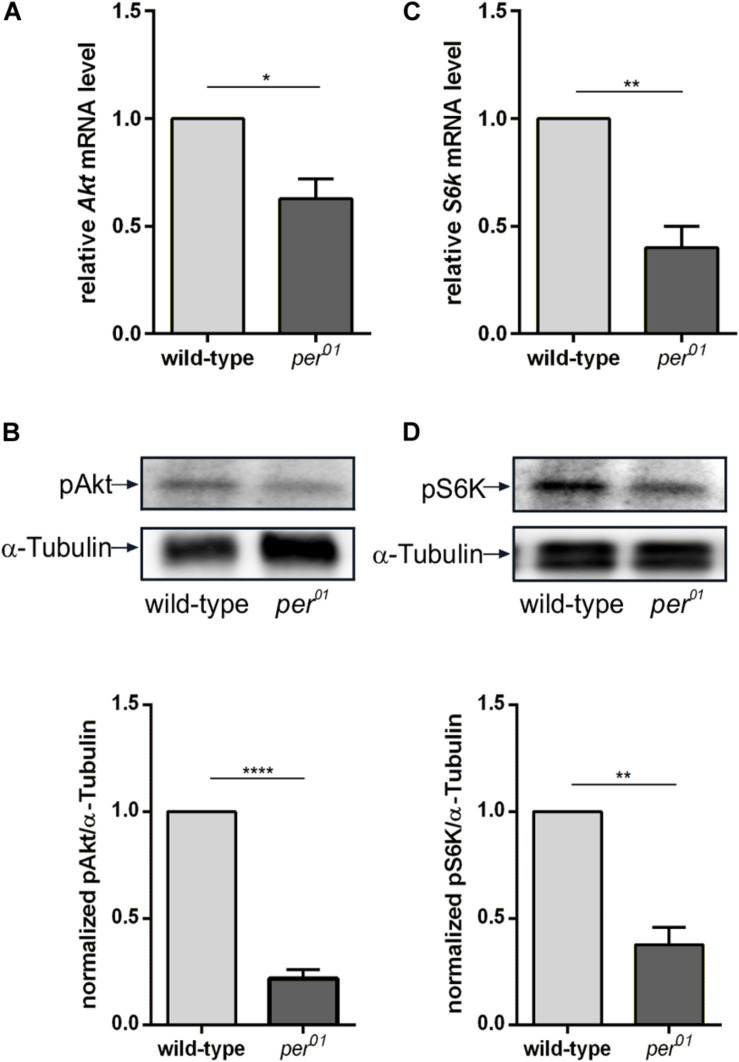
Clock dependent transcription and phosphorylation of Akt and S6K. **(A,C)** RT-qPCR analysis of *Akt* **(A)** and *S6k* **(C)** transcription in 3rd instar larval brains from wild type and *per*^01^. Each bar represents the mean ± standard error of the mean (SEM) obtained from three biological replicates, each repeated in triplicates. As an internal control, *rp49* was used (^∗^*p* < 0.01 and ^∗∗^*p* < 0.001, respectively). **(B,D)** Representative Western blots from 3rd instar larval brain lysates probed for pAkt **(B)** and pS6K **(D)**. α-Tubulin was used as a loading control. Graphs represent measurements of pAkt and pS6K phosphorylation level out of three biological replicates normalized to α-Tubulin (mean ± SEM) (^****^p < 0.00001 and ^∗∗^*p* < 0.001).

We also analyzed the regulatory effect of the circadian clock on gene and protein expression levels of Akt and S6K. Similar to the DD condition, we did not observe differences in the Akt protein level between wild type and *per*^01^ (Supplementary Figure 5). In contrast to the DD conditions, the RT-qPCR analysis showed that disruption of the circadian clock in *per*^01^ resulted in reduced expression of *Akt* and *S6k* mRNAs (Figures 6A,C). In addition, phosphorylation of Akt and S6K were more severely reduced in *per*^01^ mutant flies in comparison to the DD condition (Figures 6B,D).

These results indicated that activation of two important components of the InR/TOR signaling pathways is under influence of light and the circadian clock. The impairment of signaling by the reduction of phosphorylation of Akt and S6K might lead to cell growth and proliferation suppression.

## Discussion

### Regulation of Neuroblast Size and Proliferation by Light and the Circadian Clock

Metabolic homeostasis relies on accurate circadian timing (reviewed in Green et al., 2008; Eckel-Mahan and Sassone-Corsi, 2013; Maury, 2019). A disrupted circadian clock causes severe disturbances in rhythmic gene expression (reviewed in Bellet and Sassone-Corsi, 2010; Hughes et al., 2012), many of them being involved in metabolism (reviewed in Panda et al., 2002). Furthermore, environmental factors such as light, temperature, or nutrition modulate circadian rhythmicity and synchronize the endogenous clock with the environment. Animals with a disrupted circadian system, caused by exposure to shifted light/dark cycles, show various pathological symptoms including metabolic deficits (Marcheva et al., 2010; Barclay et al., 2012; Mota et al., 2017; Maury, 2019).

The embryonic and postembryonic waves of neurogenesis in *Drosophila* are separated by a quiescence phase. Release from quiescence requires enlargement of NBs in a nutrition dependent manner before they initiate proliferation. Postembryonic NBs grow and regain their size between each round of division until they shrink again and exit cell cycle at the late larval and pupal stages (Ito and Hotta, 1992; Britton and Edgar, 1998; Truman and Bate, 1988; Maurange et al., 2008; Chell and Brand, 2010; Siegrist et al., 2010). In this study we showed that NB size and proliferation require both clock function and light information. Under DD conditions or absence of a functional clock (*per*^01^), a NB size defect was observed throughout larval development with stronger defects at later larval stages (Figure 1E). Larvae with a disrupted clock grown under DD condition showed an even more pronounced effect on NB size, which argues for an at least partially independent influence of the circadian system and light on NB growth (Figures 1D, 2B).

Given the correlation between increase of NB size and proliferation at the beginning and decrease at the end of the larval phase of neurogenesis in wild type animals (reviewed in Homem et al., 2015), we also expected to see a proliferation defect in the *per*^01^ mutant and animals grown under DD condition. Although both experimental groups showed a significant reduction in NB size already in 1st instar larval brains, the percentage of metaphase NBs remained at wild type levels and dropped not before 3rd larval instar (Figure 2A). One explanation is that all NBs enter the cell cycle after release from quiescence as in wild type, but the progressive reduction in NB size limits NB proliferation when they fall below a critical threshold size. An alternative explanation considers that reactivation/growth of G0 arrested NBs takes places until 48 h ALH (Otsuki and Brand, 2018), whereas our analysis of 2nd instar larvae was performed at 36 h ALH. At this time point, proliferation was apparently normal, but we cannot exclude the possibility that reactivation of remaining G0 arrested NBs between 36 and 48 h ALH was disturbed, causing the observed decrease of metaphase NBs in 3rd instar larval brains from *per*^01^ and DD kept animals.

A direct effect of light and the endogenous clock on the number of progeny cells generated from a single NB was demonstrated for mushroom body NBs and Type II NBs (Figures 3D,F). Interestingly, neither mushroom body NB derived GMCs nor Type II NB derived mINPs showed cell size defects (Supplementary Figure 2) arguing for a rather cell-type specific effect of light and the circadian clock on cell size control. Despite the reduced number of progeny cells generated from both types of NBs, an influence on final adult brain size was not evident (Supplementary Figure 3B). However, 3rd instar larval brain size of animals grown under DD condition was significantly reduced in comparison to LD conditions and the *per*^01^ mutant (Supplementary Figure A). Because 3rd instar larvae were chosen at the same developmental stage just before pupation, brain development under DD conditions is apparently delayed at this stage, and this phenotype becomes at least largely rescued during pupal stages. However, as one caveat, small changes in neuron numbers might not lead to measurable adult brain size differences in our way of analysis. Therefore, it will be necessary to perform labeling and cell counting of neurons derived from individual NB to clarify whether any structure of the adult central brain is affected under DD conditions or in the *per*^01^ mutant.

Neuroblast reactivation requires nutritional input sensed by the fat body, which in turn activates NB ensheathing glial cells by fat body derived signals (FDS) to produce Insulin-like peptides (ILP). Reactivation of NBs requires ILP signals and amino acid uptake, which stimulate the InR/TOR signaling pathways to trigger the first growth of the neuroblast before they resume proliferation (Chell and Brand, 2010; Sousa-Nunes et al., 2011). Additionally, glial cells also express Activin-like peptides (ALPs), which have a mitogenic effect on NBs and are important for stimulating NB division (Zhu et al., 2008). It will be of interest to find out whether expression of ILPs and ALPs are affected under DD condition and in clock mutants.

On the other hand, the peripheral clock in the fat body plays an essential role in feeding rhythmicity and energy storage, thus being responsible for the homeostatic control of the organism. Additionally, light also drives feeding rhythms independent of clock function (Xu et al., 2008). Thus, a major interest would be to elucidate the requirement of the peripheral fat body clock in the regulation of NB growth and proliferation.

### Coordination of Cellular Growth Regulatory Pathways by Light and the Circadian Clock

Neuroblast must gain appropriate mass and size before division, which directly correlates with a high demand for proteins. Major steps for protein biosynthesis are ribosomal RNA synthesis via rDNA transcription, ribosome biogenesis, translation, and post-translational modification. In consideration of the high metabolic costs of RNA synthesis and ribosome biogenesis, protein biosynthesis represents one of the main metabolic activities in growing and dividing cells. In mammals, the circadian clock coordinates ribosome biogenesis on the transcriptional and translational levels (Jouffe et al., 2013). The signaling pathways regulating these processes are rhythmically activated in a clock-dependent manner. Interestingly, translated mRNAs are mostly involved in ribosome biogenesis (Jouffe et al., 2013). The nucleolus is the site where RNA synthesis and ribosome biogenesis take place, thus the size of the nucleolus correlates with cell growth ability. Our observations that flies under DD condition and flies with depleted clock function have reduced nucleoli (Figures 4B,C) indicated that the observed growth and proliferation defect could be due to a failure of sufficient protein biosynthesis.

The major growth regulatory pathway, InR/TOR signaling, is highly conserved between different organisms (reviewed in Hietakangas and Cohen, 2009). Most of the genes regulated by the TOR signaling pathway are involved in rDNA transcription, ribosome biogenesis, and translation initiation (Guertin et al., 2006; Grewal et al., 2007). Furthermore, *Drosophila* larvae deficient for TOR show a reduced nucleolar size and a developmental arrest (Zhang et al., 2000). The observed cell size phenotype raised the question, whether light and the circadian clock regulate cell growth through modulation of InR/TOR signaling.

Target of rapamycin signaling is promoted by activated Akt kinase as a downstream target of InR (Gao and Pan, 2001; Inoki et al., 2002; Potter et al., 2002). Independent from InR, the TOR pathway becomes activated through nutritional or amino acid sensing mechanisms (Colombani et al., 2003; Kim et al., 2008; Shim et al., 2013). In both cases, cellular growth is stimulated by phosphorylation of TOR downstream effector proteins. We focused on protein kinase Akt as an upstream regulator of TOR and on S6K as a major downstream effector protein kinase. S6K phosphorylates and thereby regulates several proteins essential for mRNA translation initiation and translation efficiency. Additionally, activated S6K plays a role in small ribosome biogenesis by phosphorylation of ribosomal protein S6 (reviewed in Hietakangas and Cohen, 2009; Saxton and Sabatini, 2017).

Here we showed that *Akt* and *S6k* gene expression in 3rd instar larval brains were not rhythmic and total Akt and S6K protein levels did not differ significantly between wild type grown under LD and DD within a 24 h period (Figures 5A,C). However, the kinase activities of Akt and S6K in brains were significantly reduced at several time points when animals were kept in DD (Figures 5B,D). Disruption of the circadian clock in *per*^01^ animals had an even stronger negative effect on Akt and S6K activity (Figures 6B,D), but also on their gene expression levels (Figures 6A,C). These observations indicate that the decreased NB size might be caused by disturbed Akt activation of TOR resulting in impaired S6K signaling and decreased translation initiation and/or small ribosome subunit biogenesis.

Studies in mammals showed that TOR signaling and the light entrainable circadian clock reciprocal regulate each other, and also an independent food anticipatory clock has an impact on TOR signaling (Cao et al., 2011, 2013; Khapre et al., 2014). Similarly, the peripheral clock in the fat body of flies can be synchronized independently of the central clock by light and via time-restricted feeding (Xu et al., 2011). On the other hand, increasing Akt or TOR activity levels lengthens the circadian period (Zheng and Sehgal, 2010). These examples illustrate the complex interactions between central and peripheral clocks, their mutual relationship with TOR signaling, the impact of environmental factors such as periodic food availability on behavior and physiology, and vice versa the influence of metabolic dysregulation on rhythmic behavior.

The survival rate and fitness of an organism are regulated by adjusting developmental growth, metabolism, and behavior to environmental changes. Our current findings provided evidence that TOR signaling is influenced by clock dependent mechanisms and light. Natural light is important for a living organism to generate physiological and behavioral traits synchronized not only to light-dark daily rhythm but also to seasonal changes. Hence, light intensities as well as photoperiodicity might have an effect on neural circuit formation by affecting neural stem cell growth and proliferation.

## Data Availability Statement

All datasets presented in this study are included in the article/Supplementary Material.

## Author Contributions

AH designed the research. ED and AH performed the research and prepared the figures. ED, AH, and PM analyzed the data. AH and TR wrote the manuscript with support of all other authors. All authors contributed to the article and approved the submitted version.

## Conflict of Interest

The authors declare that the research was conducted in the absence of any commercial or financial relationships that could be construed as a potential conflict of interest.
