# Supplementary material for: Light Stimuli and Circadian Clock Affect Neural Development in Drosophila melanogaster
Source: Front Cell Dev Biol. 2021 Mar 5;9:595754. doi: 10.3389/fcell.2021.595754 (PMC7982892; doi:10.3389/fcell.2021.595754)
Supplement: Supplementary file 1 [file Data_Sheet_1.docx]

**Supplementary figures**

**

**

**Supplementary Figure S1. The effect of the circadian clock and light on the developmental timing.** The time from 1^st^ instar larvae till pupation was similar for all genotypes with an exception of day 5, where animals grown under DD condition showed a slight delay.

**
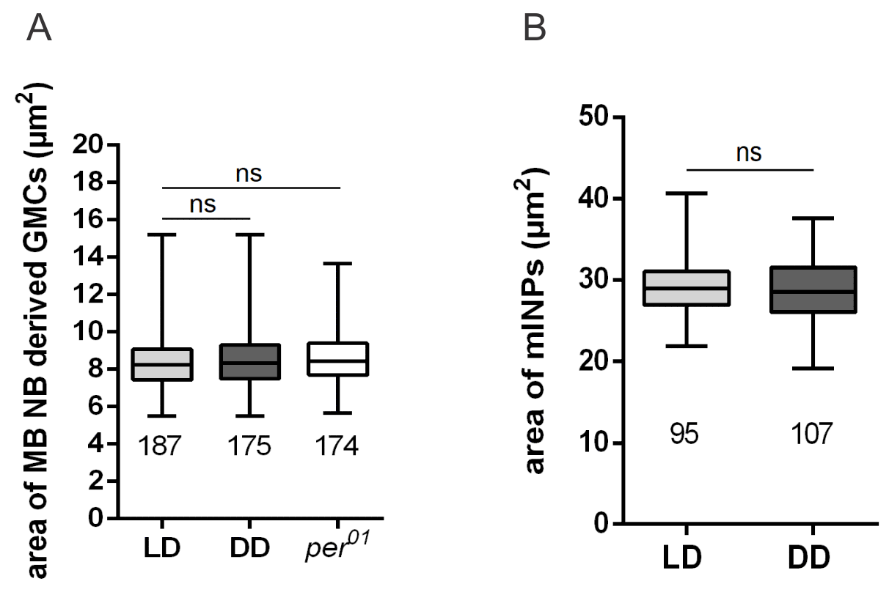
**

**Supplementary Figure S2. Light and the circadian clock have no effect on mushroom body NB derived GMC (A) and Type II NB derived mINP sizes (B).** At least 10 brains were analyzed for each genotype and different light conditions. The number of measured GMCs and mINPs are indicated below of each box plot.


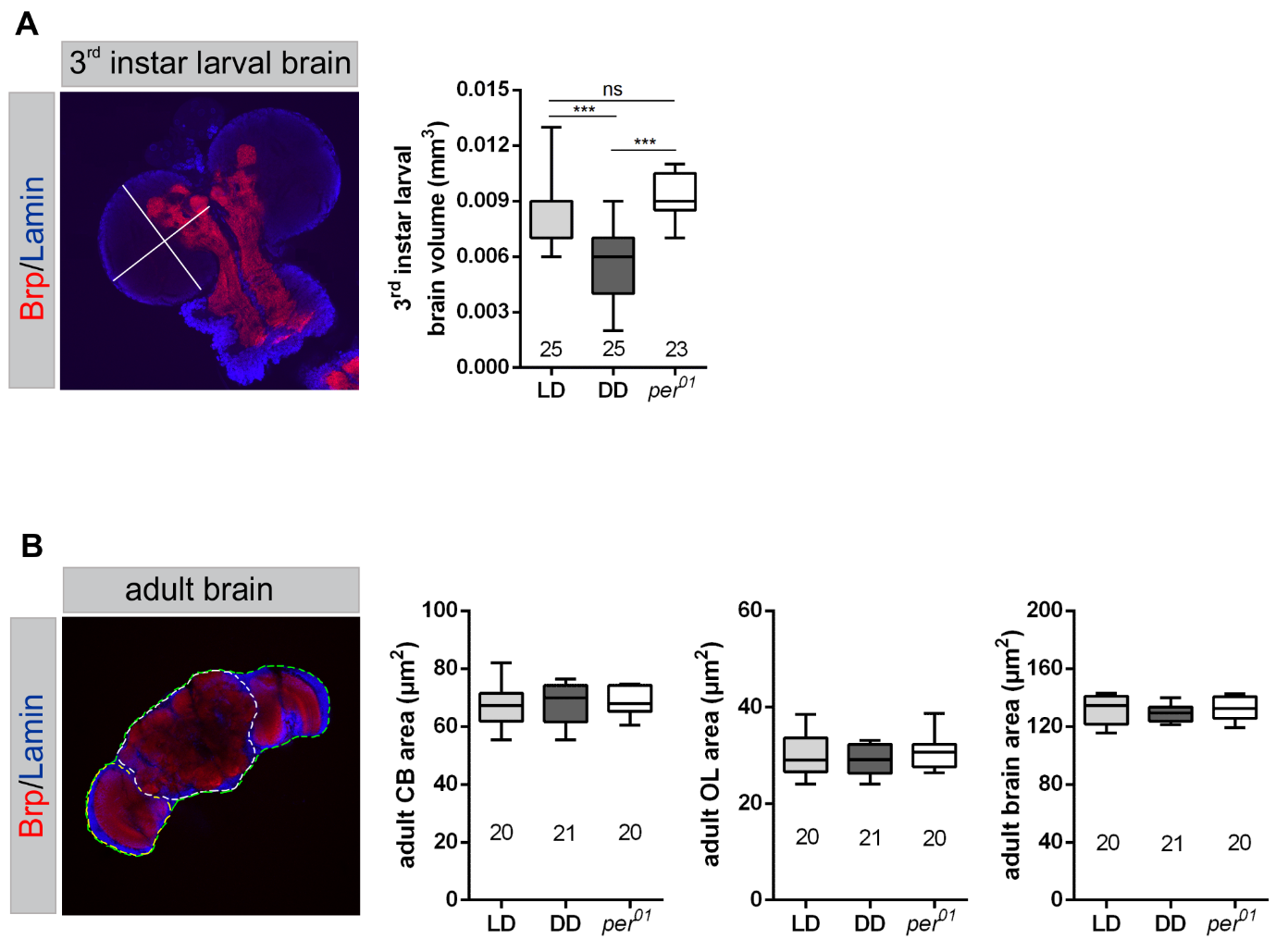


**Supplementary Figure S3. Influence of light and the circadian clock on brain size in larvae and adult.**  Representative images of brains from a late wandering 3^rd^ instar larvae **(A)** and a newly hatched adult fly **(B).** Brains were immunostained for the synaptic protein Bruchpilot (Brp, red) and Lamin (blue) to mark nuclear membranes. **(A)** Wild type larval brains grown under DD condition were significantly smaller compared to brains from wild type (LD) and *per^01^* mutants (p < 0.0001). **(B)** Measurements of the areas of the central brain (white dashed line), optic lobe (yellow dashed line) and the whole adult brain (green dashed line) showed no differences between the experimental groups. The number of analyzed brains are indicated below of each box plot.

**
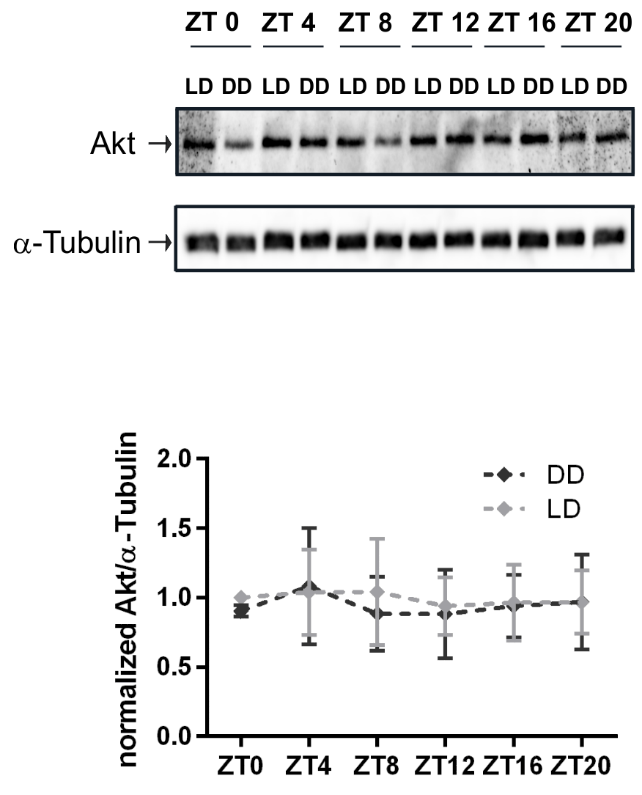
**

**Supplementary Figure S4. Light-dependent expression of Akt.** Representative Western blot showing Akt expression in brain extracts from wild-type 3^rd^ instar larvae grown under LD and DD conditions at different time points during 24 hours. α-Tubulin was used as a loading control. The graph below shows quantification of Akt expression levels out of seven biological replicates, normalized to α-Tubulin (mean±SEM).


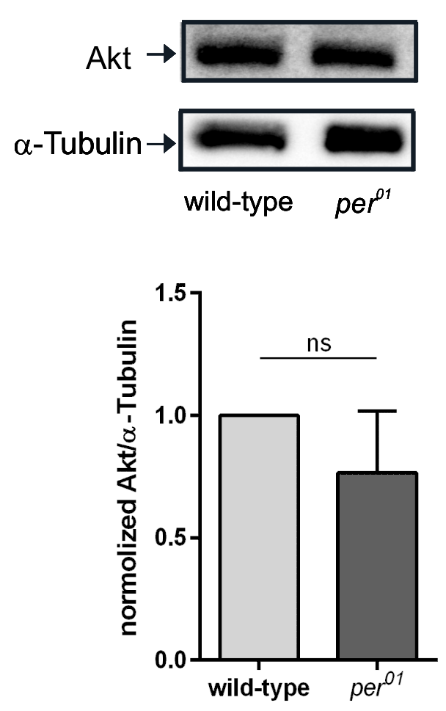


**Supplementary Figure S5. Clock dependent expression of Akt.** Representative Western blot of Akt expression in brain extracts from wild-type and *per^01^* 3^rd^ instar larvae at ZT0. α-Tubulin was used as a loading control. Graphs show quantification of Akt expression levels out of three biological replicates, normalized to α-Tubulin (mean±SEM).
